# The Synthesis of a New Glycoluryl–Melamine–Formaldehyde Polymer under the Action of HEDP and the Investigation of the Content of Methylol Groups and Free Formaldehyde

**DOI:** 10.3390/polym16202877

**Published:** 2024-10-12

**Authors:** Nurdana Kanasheva, Arthur Ukhov, Victor S. Malkov, Alexander Gubankov, Samal Sergazina, Manar A. Issabayeva, Togzhan Mashan, Ainagul Kolpek, Roza Ryskaliyeva, Abdigali Bakibaev, Rakhmetulla Yerkassov

**Affiliations:** 1 Department of Chemistry, L.N. Gumilyov Eurasian National University, Astana 010008, Kazakhstan; 2 Faculty of Chemistry, National Research Tomsk State University, 634028 Tomsk, Russia; 3 Department of Chemistry and Biotechnology, Sh. Ualikhanov University, Kokshetau 020000, Kazakhstan; 4 Department of Chemistry and Chemical Technologies, Toraighyrov University, Pavlodar 140008, Kazakhstan; 5 Faculty of Chemistry and Chemical Technology, Department of General and Inorganic Chemistry, Al-Farabi Kazakh National University, Almaty 050000, Kazakhstan

**Keywords:** glycoluryl, glycoluryl–melamine–formaldehyde resins, HEDP, free formaldehyde in polymers

## Abstract

This study outlines a method for preparing a complex involving glycoluril and melamine (GU-ME). The structure of the resultant complex was analyzed using IR and NMR spectroscopy. In the subsequent phase, the polymer GUMEFA was derived from the resultant complex, employing hydroxyethylidene diphosphonic acid (HEDP) as a sustainable plasticizer, with a proposed chemical mechanism for its formation. The molecular weight of the resulting GUMEFA was analyzed, and the formation chemistry was proposed. GUMEFA was characterized, and its free formaldehyde and methylol group contents were investigated. It was observed that GUMEFA prepared with HEDP contained approximately 1.15–1.34 wt.% free formaldehyde and 1.56–0.54 wt.% methylol groups. These findings provide valuable insights for developing resins of different compositions and applications, thereby paving the way for producing composite materials with tailored properties.

## 1. Introduction

In practical human applications, the diverse range of polymer substances includes crucial resins like phenol–formaldehyde, urea–formaldehyde, and melamine–formaldehyde resins (MFRs). MFRs are utilized both independently and in combination with urea–formaldehyde resin to enhance their strength and fire resistance within cellulose-containing composites. Research is also advancing on amine-containing, phenolic, and isocyanate resins [1]. The latest thermoset adhesives are preferred for their economic benefits, offering versatile properties in their cured state with rapid curing capabilities. Urea–formaldehyde (UF) resins stand out as the most significant adhesives due to their cost-effective raw materials, fast curing times, high dry bond strength, and transparent glue lines. They are predominantly used in manufacturing wood-based materials such as particleboards or medium-density fiberboards [2]. For applications in high-humidity conditions, UF resins are typically modified with more expensive compounds like melamine, phenol, or resorcinol [2]. The choice of final adhesive composition depends on specific requirements for the wood material, including the desired strength properties, expected resistance to moisture, production costs, and targeted formaldehyde emissions.

In industry, modifications are commonly applied, such as incorporating urea into an aqueous solution or powder form, organic amines, resin scavengers, sulfites, functionalized paraffin waxes, and porous materials like pozzolan and charcoal [3]. Significant reductions in formaldehyde emissions can be achieved by adding sodium metabisulfite to the resin, introducing a tannin solution into urea–formaldehyde resins, or utilizing different starch derivatives [4,5].

Of particular interest is the direct modification of resin structures to enhance their key characteristics. The literature extensively discusses numerous resins where compounds containing amino groups such as urea (and its derivatives), melamine (and its derivatives) are combined with the carbonyl compound formaldehyde [6]. Furthermore, patents detail methods and formulations for urea–formaldehyde resins [7] and urea–melamine–formaldehyde resins [8], which are widely used in manufacturing various composite materials like particleboards, fiberboards, and plywood. Another structural modification option for urea–formaldehyde resins involves glycoluril (GU). There are documented instances where glycoluril is utilized during curing as a formaldehyde-binding agent [9,10], suggesting its ability to undergo condensation reactions with formaldehyde. Additionally, a mixture of melamine and glycoluril has been used as a fire retardant in thermoplastics [11]. These combined chemical properties position glycoluril as a promising cross-linking agent in polycondensation reactions involving formaldehyde-containing resins [11].

Glycoluril–melamine–formaldehyde resins are synthesized through the condensation of urea and/or melamine with formaldehyde in a neutral or slightly alkaline environment, followed by thermal and/or acid curing. Initially, the polycondensation reaction produces water-soluble oligomeric products. In the subsequent stage, these oligomeric fragments undergo cross-linking to form a network polymer. This process involves the formation of methylene bridges, resulting from the interaction of methylol groups with the hydrogen atoms of NH groups, and ether bonds between methylol groups, creating a spatially structured polymer [12]. Depending on the reaction conditions, the resulting polymer may have linear, cyclic, or spatial fragments [13].

Uncured resins are viscous liquids that are either yellow or colorless. Once cured, these resins do not melt, dissolve, or soften. However, due to high shrinkage, cured resins often crack [14]. If the reaction temperature is too high or if there is long-term storage, low pH, or prolonged polycondensation reactions, poorly soluble and water-insoluble compounds can form, precipitating as a white sediment. Once precipitation occurs, the reaction ceases. This precipitation is also linked to a decrease in the content of free formaldehyde in the solution due to the Cannizzaro reaction, which leads to the formation of formic acid [15].

A significant drawback of the described resins and materials based on them is their extremely low moisture resistance. Hydrolytic action leads to the destruction of the polymer chains, and the presence of methylene ether bonds results in the release of formaldehyde and other toxic compounds into the environment [16]. Another issue is the fragility of pure MFR, making it unsuitable for use without modifications. Additionally, medium-term storage of the resin presents challenges. These combined disadvantages significantly limit the potential applications of MFR. Despite efforts, it has not been possible to eliminate the internal stresses in the resin caused by shrinkage while simultaneously improving its strength properties. Consequently, resins modified with glycoluril are attracting increasing interest from researchers.

Additionally, the plasticization of melamine–glycoluril–formaldehyde resins is achieved using the Mannich reaction in the presence of strong inorganic and organic acids, such as hydrochloric, sulfuric, and formic acids. This requirement imposes restrictions on the use of such polymers due to the high toxicity and hazards associated with these acids during their emission. Recently, studies have found that 1-hydroxyethylidene diphosphonic acid (HEDP) serves as an effective "green" catalyst in various chemical processes [17]. For instance, HEDP is a convenient catalyst for forming dihydropyrimidones from carbonyl compounds with active methylene groups, urea, and aldehydes, both under traditional [18] and microwave synthesis conditions [19], and in the synthesis of glycoluril and its derivatives [20,21,22,23]. This makes HEDP an attractive plasticizer for producing resins for various applications.

## 2. Materials and Methods

### 2.1. Synthesis of Glycoluril–Melamine–Formaldehyde Resin (GUMEFA)

In the initial stage, a glycoluril (LLC, Novokhim, Tomsk Oblast, Russia) and melamine (JSC AMK-group, Barnaul, Russia) complex (GU-ME) was prepared following the method described in [24]. In the subsequent stage, this complex was plasticized with formaldehyde (Scharlab, Barcelona, Spain) in the presence of HEDP (VitaChem, Moscow, Russia).

#### 2.1.1. Synthesis of the Glycoluril–Melamine Complex (GU-ME)

In a round-bottom flask, 0.61 g (0.0043 mol) of glycoluril, 2.15 g (0.0171 mol) of melamine, and 55 mL of distilled water were combined and stirred at 90 °C for 2.5 to 3 h. The solution was then filtered through a Buchner funnel without prior cooling, and the precipitate was dried to a constant weight. The dried precipitate was used directly for the synthesis of GUMEFA. The product yield was 1.4 g.

#### 2.1.2. Synthesis of GUMEFA via Plasticization with Hydrochloric and HEDP Acids 

In the subsequent stage, the polymer GUMEFA was synthesized from the GU-ME complex through plasticization, which was catalyzed by varying concentrations of hydrochloric acid (Sigma Tec, Moscow, Russia) and hydroxyethylidene diphosphonic acid (HEDP) (VitaChem, Russia). To 8.5 mL of a 36.6 % aqueous formaldehyde solution (0.1 mol), 0.3 mL of a 25 % aqueous ammonia solution (0.0044 mol) and 1.4 g of the GU-ME complex were added and stirred at 60–65 °C until the mixture was homogenized. Then, 3.1 g (0.0246 mol) of melamine was added and stirred until the mixture became homogeneous. After homogenization, the resin was cured using different methods:

- Method 1: by adding HEDP solution (1 mL of HEDP solution with a concentration of 0.5 g per 1 mL of water), with a plasticization time from 20 h to 24 h [25];
- Method 2: by adding crystalline HEDP acid (0.5 g of HEDP acid), with a plasticization time from 5 to 10 min [25];
- Method 3: an experiment conducted without using a plasticizer, serving as a control; with the plasticization period lasting three days.

Additionally, for the control and comparison, a polymer was synthesized using hydrochloric acid following a well-established method [26]. The following plasticization conditions were selected:

- Method 4: by adding hydrochloric acid solution (1 mL of 8% diluted solution), with the time for complete plasticization being from 20 h to a day;
- Method 5: by adding concentrated hydrochloric acid (0.2 mL of 36% concentrated acid), with a plasticization time from 5 to 10 min.

In all cases, the polymer yield was about 5 g.

### 2.2. Physicochemical Research Methods

#### 2.2.1. IR Spectroscopy

The samples were analyzed using Fourier-transform infrared (FTIR) spectroscopy with a Nicolet 6700 IR spectrometer from Thermo Fisher Scientific (Waltham, MA, USA). Attenuated total internal reflection (ATR) was employed to study the samples in the spectral range of 400 to 4000 cm^−1^, with a resolution of 4 cm^−1^. The reflection spectra obtained were then converted into absorption spectra using the Kubelka–Munk transformation.

#### 2.2.2. NMR Spectroscopy

The NMR spectra of the synthesized compounds were acquired using a Bruker Avance 400 III HD NMR spectrometer (Billerica, MA, USA), operating at 400 MHz for hydrogen nuclei and 100 MHz for carbon-13 nuclei, in a CDCl_3_ solution at 25 °C.

#### 2.2.3. Melting Temperature 

The melting point was measured using an M-560 melting point analyzer (Buchi, Flawil, Switzerland), with a heating rate of 0.1 °C/s. The obtained results were compared to the data reported in the literature. 

#### 2.2.4. Gel Permeation Chromatography

Measuring instruments and equipment: Agilent 1260 liquid chromatograph with a refractometric detector (Agilent Technologies, Santa Clara, CA, USA); software (Agilent ChemStation Rev. B.04.03 16) for GPC analysis result processing, “Agilent GPC Addon Rev.B.01.02”; gel permeation chromatography column PLgel 5 µm MIXED-D 300 × 7.5 mm^2^; variable-volume single-channel pipette dispenser DPOP-1–100–1000 “Lenpipet-Light”, with a volume range up to 1000 µL.

Reagents and materials: stabilized chloroform for HPLC; polystyrene standards for calibration, such as Calibration kt or PL2010-0100; chromatographic vials with screw caps.

Measurement conditions: detector: refractometric; column: PLgel 5 µm MIXED-D 300 × 7.5 mm^2^; column temperature: 30 °C; isocratic elution; flow rate: 1 mL/min; mobile phase: chloroform; analysis time: 15 min; sample injection volume: 50 µL.

Sample preparation: a 1.0 mL aliquot of the sample was transferred to a 2 mL vial for analysis.

#### 2.2.5. Determination of Methylol Group Contents 

The method involves oxidizing methylol groups and free formaldehyde (“total” formaldehyde) with iodine in an alkaline medium. Subsequently, the excess iodine is titrated with a sodium thiosulfate solution. By subtracting the content of free formaldehyde from the resulting total formaldehyde, the content of methylol groups is determined [27]. 

A sample of the analyzed GUMEFA, weighing 0.2380 g, was placed in a conical flask and dissolved in 30 mL of distilled water with stirring. To this solution, 25 mL of 0.1 N iodine solution and 10 mL of 1 N potassium hydroxide solution were added. The mixture was stirred and left in a dark place for 15 min, with the flask tightly closed with a stopper. Then, 15 mL of 1 N sulfuric acid solution was added to the flask, mixed, and after 5 min, the contents were titrated with 0.1 N sodium thiosulfate solution. When the solution turned straw yellow, a starch solution was added, and the titration continued until the blue color disappeared. A control experiment was conducted simultaneously in the same manner without the analyzed sample.

The content of methylol groups, x (in %), was calculated using the following Equations (1) and (2):

x = x_1_ − x_2_ (1)

(2) $$x_{1}=\frac{(V_{1}\;-\;V_{2})\;\times \;100\;\times \;0.0015\;\times \;100}{m}$$

where x_1_ is the total content of methylol groups and free formaldehyde in terms of formaldehyde, which is determined using the iodometric method, %; x_2_ is the content of free formaldehyde in the sample, %; V_1_ and V_2_ are volumes of 0.1 N sodium thiosulfate solution used for the titration of control and working solutions, respectively, mL; 0.0015 is the amount of formaldehyde, 1 mL of which contains exactly 0.1 N sodium thiosulfate solution, g; and m is the sample weight, g. 

The test result is determined as the arithmetic mean of the results from two parallel measurements, with a permissible discrepancy not exceeding 3%.

#### 2.2.6. Determination of Formaldehyde Content 

The concentration of formaldehyde in the polymer was analyzed using two methods. The first method was spectrophotometric, being based on the interaction of formaldehyde with acetylacetone in ammonium acetate, producing a yellow-colored compound [28]. The second method was fluorimetric, which involved the formation of a luminescent compound when formaldehyde reacted with 1,3-cyclohexanedione in the presence of ammonium ions [28].

Spectrophotometric method: for this method, a PE-5400UF spectrophotometer (EKROSHIM LLC, St. Petersburg, Russia) was used; spectral range: 190–1000 nm; spectral slit width: 4 nm; wavelength setting error: no more than ±1 nm; wavelength setting reproducibility: ±0.5 nm; limits of permissible absolute error when measuring spectral coefficients of directional transmittance: no more than ±0.5%T (315–1000 nm) and ± 1.0%T (190–315 nm); measuring range:

1. Optical density: from 3.000 to 0.000;
2. Directional transmittance: from 0.0 to 100.0%.

Light source: deuterium and halogen lamps. 

Preparation of acetylacetone–ammonia reagent: A total of 15 g of ammonium acetate was added to a 100 mL volumetric flask and dissolved in a small amount of distilled water. Then, 0.3 mL of glacial acetic acid and 1 mL of acetylacetone were added. The flask was filled up to the 100 mL mark with distilled water and mixed thoroughly.

Preparation of calibration solutions: Here, 8–9 mL of distilled water was placed into a graduated test tube with a capacity of 25 mL, and the following was added with a graduated pipette, respectively: 0.0; 0.5; 1.0; 1.5; 2.0; 4.0; 6.0; 8.0; and 10.0 mL of a working solution of formaldehyde with a concentration of 1 mg/L; then, 5 mL of acetylacetone–ammonium reagent was poured into each flask, diluted to the mark with distilled water, and mixed. The values f the mass concentrations of formaldehyde in the calibration solutions are as follows, respectively: 0.0; 0.02; 0.04; 0.06; 0.08; 0.16; 0.24; 0.32; and 0.40 mg/L. A calibration solution that does not contain formaldehyde (with a mass concentration of formaldehyde equal to zero) was used as a blank sample.

The solutions were prepared on the day they were to be used. Each prepared calibration solution and a blank sample were placed in a water bath at a temperature of 60–65 °C for 10 min. The flasks were then cooled to room temperature, either in a cold water bath or under running cold water. The optical densities of the calibration solutions and the blank sample were measured at a wavelength of 414 ± 20 nm in an optical cuvette with an absorbing layer thickness of 5 cm, using distilled water as the reference solution. Following this, the formaldehyde content in the polymer was determined.

Polymer modeling conditions: model medium: distilled water; saturation of the aqueous solution: 0.01 g: 200 mL or 0.05 g: 200 mL; temperature of the aqueous solution (60 ± 2) °C; exposure time: 1 h.

A total of 8 mL of distilled water was placed into a 25 mL graduated test tube. Using a graduated pipette, the model solution was added in the following volumes: 0.5 mL and 1.0 mL for a polymer mass of 0.05 g and 5.0 mL for a polymer mass of 0.01 g. Next, 5 mL of acetylacetone–ammonium reagent was added to each test tube, which was filled to the 25 mL mark with distilled water and mixed thoroughly. The prepared samples of the analyzed solution and the blank sample were then heated and cooled as previously described.

Processing of measurement results. The mass concentration of formaldehyde in the sample of the analyzed model solution X, mg/L, was calculated using Equation (3):

(3) $$X=\frac{(K\;\times \;A\;\times {\;V}_{C})}{V_{S}}$$

where K is the calibration coefficient. A is the difference between the optical density of the analyzed water sample and the optical density of the blank sample and is measured in units of optical density. V_C_ is the volume of the graduated test tube used to prepare the sample for measurement (in this case, 25 mL), in milliliters. V_S_ is the volume of the sample used for measurements, in milliliters.

The amount of formaldehyde, C, in grams per gram of polymer was calculated using the following Equation (4):

(4) $$C=\frac{X\;\times \;V_{M}}{1000\;\times \;m}$$

where X is the mass concentration of formaldehyde in the sample of the analyzed model solution, mg/L; V_M_ is the volume of the model solution, l; m is the mass of the polymer used to study the solution, g; and 1000 is the conversion factor in g. 

Next, Equation (5) was used to calculate a percentage of 1 g of polymer:

(5) $$C,\%=\frac{C\times 100\;\%}{1}$$

Fluorimetric method: for this method, a Fluorat-02–2M liquid analyzer (Lumex, St. Petersburg, Russia) was used; spectral range of optical radiation, nm: excitation channel 200–650; transmission channel 200–650; and registration channel 250–650; measurement ranges: mass concentration of phenol in water, mg/L: 0.01–25; sample transmittance, %: 10–90.

Preparation of a solution of 1,3-cyclohexanedione in an ammonia acetate buffer solution: Here, 10 g of ammonium acetate was dissolved in 50–60 mL of distilled water, 2.4 mL of concentrated hydrochloric acid was added, and 10 mg of 1,3-cyclohexanedione was added. Once fully dissolved, the solution was diluted with distilled water to the 100 mL mark in a volumetric flask.

The calibration solutions were prepared in vessels with screw caps (vials). A total of 2 mL of reagent solution was added to each of two containers. Into the first vessel, 3.0 mL of distilled water was added, and into the second vessel, 3.0 mL of a formaldehyde solution with a mass concentration of 0.5 mg/L was added. The vessels were sealed with their screw caps and then placed in a water bath and heated at 100 °C for 10 min. The solutions were cooled by placing the vessels into a glass filled with cold water. The analyzer was then calibrated by measuring the fluorescence signals of the blank sample solution and the formaldehyde solution with a mass concentration of 0.5 mg/L. Calibration was performed during the analysis of each batch of samples. 

The determination of formaldehyde in GUMEFA polymer occurred as follows: 

Polymer modeling conditions: model medium: distilled water; saturation of aqueous solution: 0.01 g: 200 mL or 0.05 g: 200 mL; temperature of the aqueous solution (60 ± 2) °C; exposure time:1 h. 

Here, 0.5 or 1.0 mL of the model solution was transferred into vessels with a screw cap (vials), distilled water was added to a volume of 3 mL, and 2 mL of reagent solution was added. Then, we proceeded with the same steps as used for the calibration solutions. 

The measurement results were processed by calculating the mass concentration of formaldehyde in the analyzed model solution sample, X (mg/L), using the following Equation (6):

(6) $$X={\;C}_{\mathrm{meas}}\;\times \;Q$$

where C_meas_ is the measured mass concentration of formaldehyde in the model solution, mg/L; and Q is the sample dilution factor.

## 3. Results and Discussion

**Figure 1:**
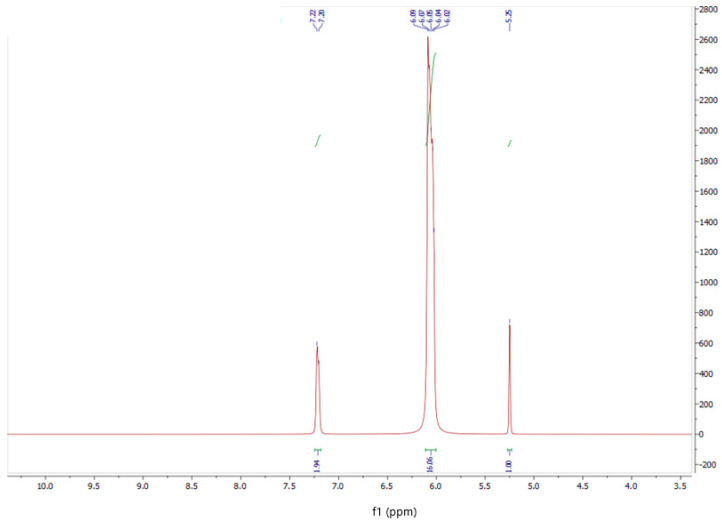
1H NMR spectrum of the obtained GU-ME complex.

**Figure 2:**
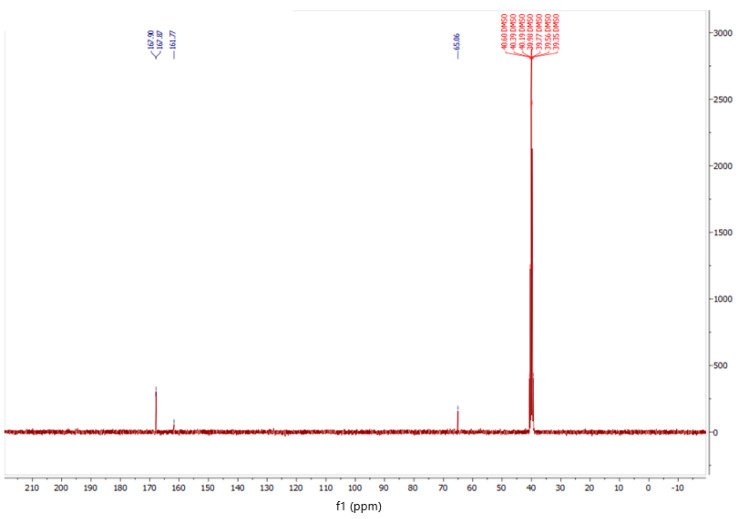
13C NMR spectrum of the obtained GU-ME complex.

In the initial phase of the study, a glycoluril and melamine complex (GU-ME) was synthesized. The composition and structure of the resulting complexes were analyzed using IR and NMR spectroscopy (Figure 1 and Figure 2).

The melting points of these complexes were 325 °C and 321 °C with decomposition. Compared to the melting temperatures of glycoluril (>300 °C with decomposition) and melamine (345 °C with decomposition), the observed temperatures clearly differ, indicating the formation of a complex. 

The analysis of the 1H NMR spectrum of the GU-ME complex reveals that the integral intensity ratio of the C-H protons of glycoluril at 5.25 ppm to the N-H protons of melamine at 6.05 ppm is 1:5. 

The obtained data are also confirmed by the 13C NMR spectrum of the obtained compound (Figure 2). At 65.06 ppm, C-H signals of glycoluryl, at 161.77 ppm, C=O of glycoluryl, and at 167.9 ppm, signals in the melamine ring are observed.

**Figure 3:**
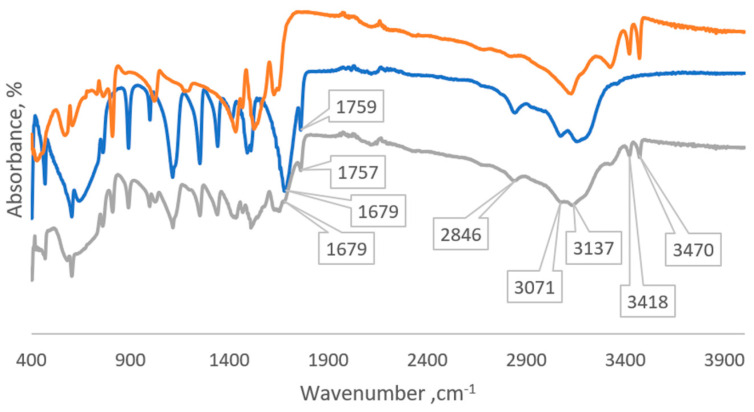
Comparative IR spectra of melamine (orange line), glycoluril (blue line), and GU-ME complex (grey line).

When examining the IR spectrum of the resulting GU-ME complex (Figure 3) and comparing it with the spectra of glycoluril and melamine, specific absorption bands are identified. These bands include those that are characteristic of glycoluril (C-H stretching vibrations at 3071 cm^−1^, N-H bending vibrations at 1757 cm^−1^, and C=O stretching vibrations at 1679 cm^−1^) and melamine (NH_2_ stretching vibrations at 3470 and 3418 cm^−1^). Additionally, there is a noticeable decrease in the intensity of the absorption bands for the carbonyl group of glycoluril and the amino groups of melamine, which is likely associated with the formation of the glycoluril–melamine complex.

**Figure 4:**
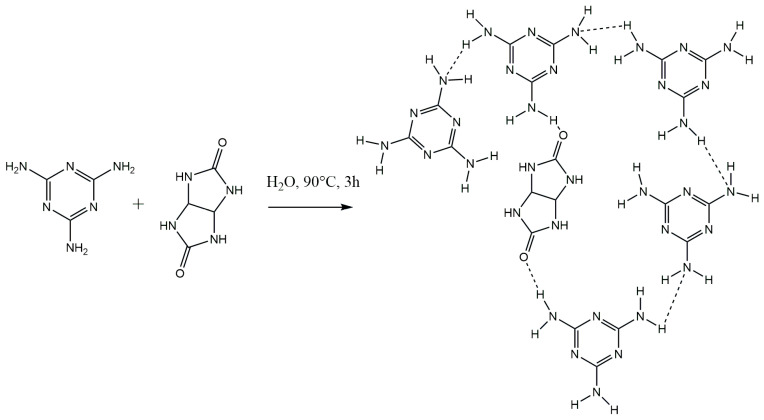
Scheme for synthesizing the GU-ME complex.

The formation of the GU-ME complex does not follow a stoichiometric process. The combination of the aforementioned facts suggests that melamine molecules coordinate not only with glycoluril but also with each other in a similar manner [24]. Based on this, a scheme for obtaining the GU-ME complex is proposed (Figure 4). These findings are supported by the literature data [24], which include examples of the formation of glycoluril–melamine complexes in varying ratios (1 mole of glycoluril to 2–4 moles of melamine).

**Figure 5:**
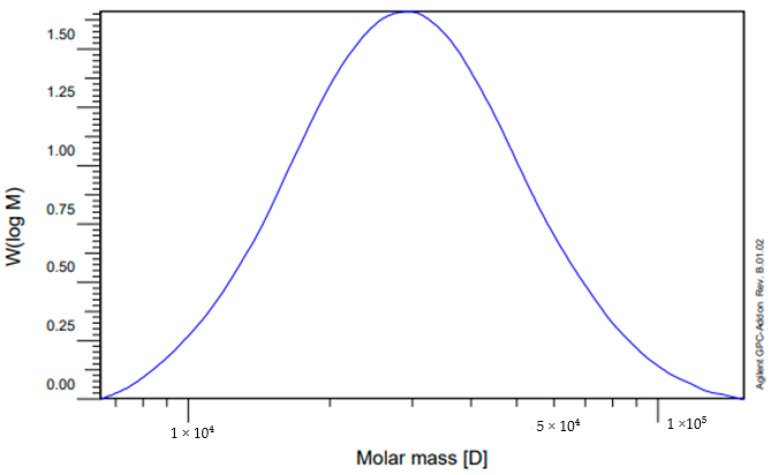
GPC analysis of GUMEFA, high-molecular-weight fraction.

In the next phase of the work, the synthesis of the polymer (GUMEFA) from the GU-ME complex was conducted, varying the concentration of the plasticizer or without using a plasticizer. It was found that the concentration of acids plays a key role in the plasticization time of the polymer. For instance, using HEDP and concentrated hydrochloric acid resulted in plasticization within about 5 min, whereas using dilute solutions extended the process up to a day. In the experiment without a plasticizer, complete plasticization of the polymer occurred within three days. The resulting polymer samples were ground in a laboratory mill to an average particle size of about 150–200 microns. GPC analysis of the resulting GUMEFA was performed to determine the average molecular weight of the polymer. This involved extracting the GUMEFA polymer with chloroform at room temperature. It was determined that the polymer consists of a high-molecular-weight fraction (Figure 5), with an average molecular weight ranging from 3.3 × 10^4^ to 4.4 × 10^4^ g/mol. The obtained polymer product was also tested for degradation at high temperatures. It was found out that GUMEFA does not have thermoplastic properties, and degradation occurs at temperatures of 345–360 °C.

**Figure 6:**
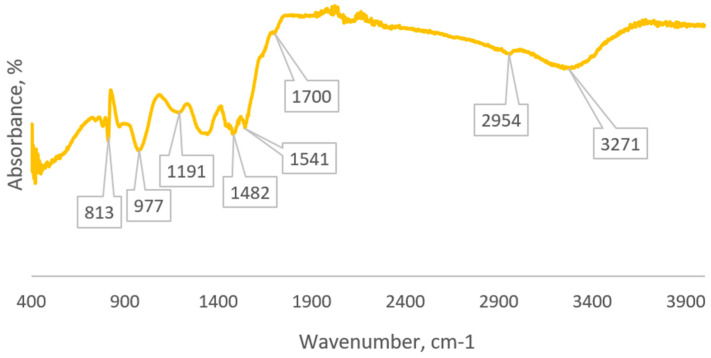
IR spectrum of GUMEFA.

In the IR spectrum of GUMEFA, both glycoluril- and melamine-related fragments are evident. The IR spectrum (crystal, cm^−1^) shows the following peaks: 3271 (NH stretching vibrations), 2953 (CH₂ stretching vibrations), 1700 (C=O stretching vibrations), 1549 (C=N stretching vibrations), 1488 (C=N stretching vibrations), 1356 (C-N stretching vibrations), 1191 (>NH stretching vibrations), and 812 (NH bending vibrations) (Figure 6).

**Figure 7:**
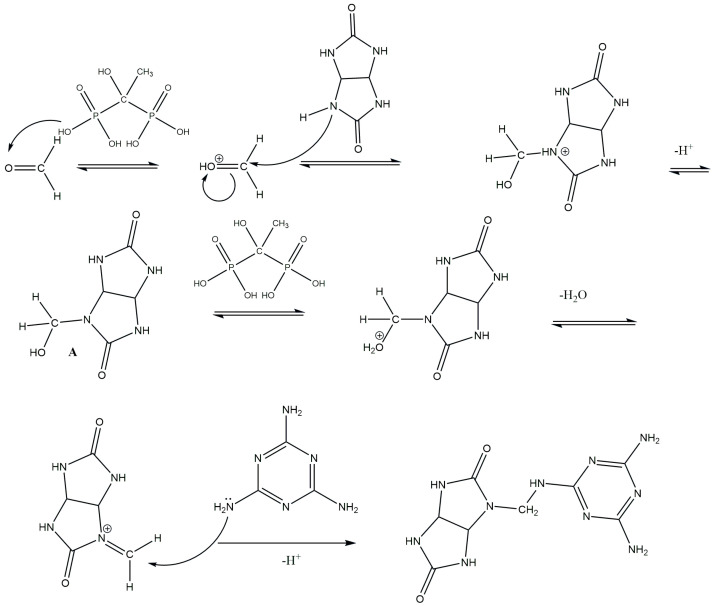
Reaction mechanism for the formation of the GUMEFA polymer under the influence of HEDP.

Based on the above information, we propose the following reaction mechanism: When glycoluryl reacts with formaldehyde in the presence of HEDP, methylol groups are formed through the Mannich reaction, as described in [29]. In the subsequent stage, these methylol groups react with melamine to create methylene bridges. Additionally, if the reaction between glycoluryl and melamine does not take place, free methylol groups are generated in both glycoluryl and melamine (Figure 7, product A), which can later eliminate formaldehyde under environmental conditions [30].

**Figure 8:**
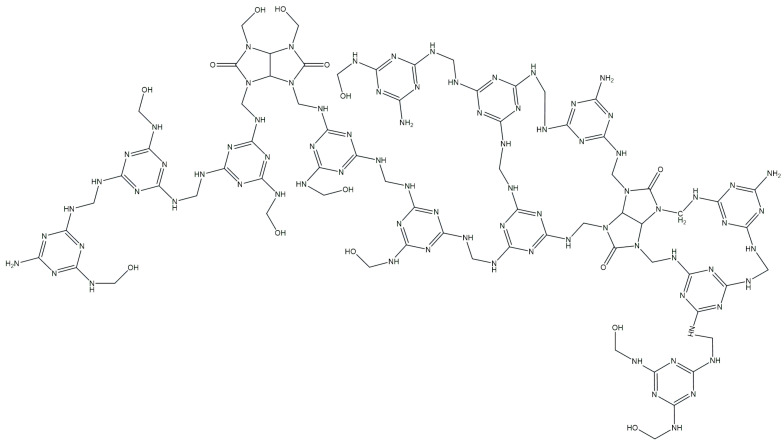
Proposed structure of the resulting GUMEFA.

Additionally, melamine can cross-link with itself through a similar mechanism, resulting in a branched melamine–melamine structure (Figure 8). From this, a polymer structure has been proposed (Figure 8). According to the proposed reaction mechanism, methylol groups form in glycoluryl (Figure 6, compound A), which may gradually release formaldehyde over time [21].

**Figure 9:**
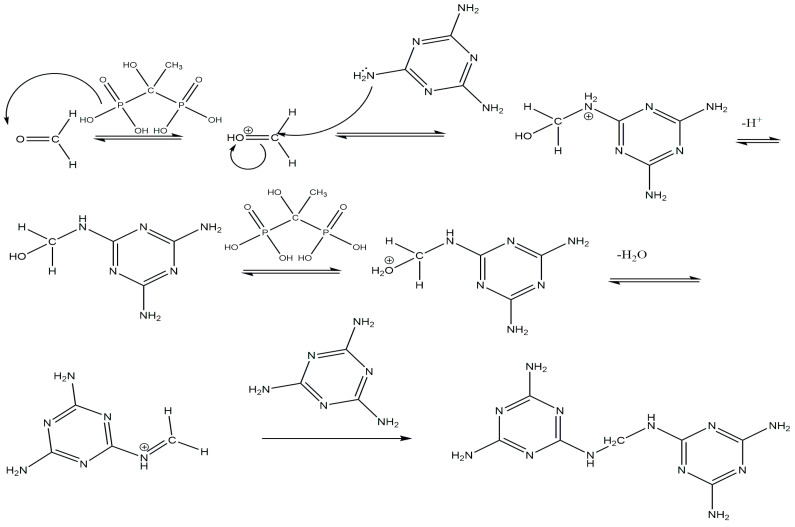
Reaction mechanism for the formation of melamine fragments in the GUMEFA polymer under the influence of HEDP.

It is known that glycoluryl readily forms tetramethylolglycoluril [31], and similarly, melamine forms trimethylolmelamine [30]. This results in competitive interactions between melamine and formaldehyde in the reaction mixture (Figure 9). These reactions occur simultaneously and in parallel, leading to the development of a network structure in GUMEFA.

Next, the free formaldehyde content in GUMEFA was analyzed using spectrophotometric and fluorimetric methods. The study revealed that the highest formaldehyde content was present in the sample produced without a plasticizer. This observation is attributed to the fact that in the absence of a plasticizer, polymerization is prolonged, allowing unbound formaldehyde to remain within the polymer without being incorporated into the structure.

In the analysis of GUMEFA produced with the plasticizer HCl, it was observed that as the acid concentration increases, the amount of free formaldehyde also rises (Table 1) by 0.14 wt.%, according to the spectrophotometric method, and by 0.12 wt.%, according to the fluorimetric method. This is explained by the fact that higher concentrations of HCl accelerate the polymerization rate, causing formaldehyde to be trapped within the polymer structure without being incorporated into it.

In the study of GUMEFA with the plasticizer HEDP, a similar trend was observed: the formaldehyde content in the polymer was higher when using crystalline HEDP compared to its solution. However, the difference between the HEDP solution and crystalline HEDP is minimal, at 0.02 wt.%. This slight variation is attributed to the selective interaction of the plasticizer with glycoluryl in the GU-ME complex.

In the next stage, both the methylol groups and formaldehyde were determined together using reverse iodometric titration. For this experiment, the GUMEFA was ground into a powder and placed in a chemical beaker. Distilled water was then added, and the mixture was maintained at 60 °C for one hour. The resulting solution was subsequently analyzed using reverse iodometric titration. The results of the titration are presented in Table 2.

According to the data in Table 2, the highest content of formaldehyde and methylol groups by weight is found in the polymer where hydrochloric acid was used as the plasticizer. This is because HCl is a much stronger acid than HEDP, leading to a rapid reaction of formaldehyde and the formation of methylol groups. The reaction rate varies with the acid concentration: it is faster with concentrated HCl and slower with diluted HCl. As the reaction with formaldehyde proceeds more quickly, methylol groups form more rapidly (Figure 6, substance A).

The low levels of formaldehyde along with methylol groups suggest that in the absence of acid, the plasticization process takes significantly longer. The methylol groups formed in this scenario quickly react with melamine to create a network structure. Additionally, the extended plasticization time in the air allows for the desorption of unreacted formaldehyde from the surface of GUMEFA. These observations are supported by the spectrophotometric and fluorimetric measurements of formaldehyde in the polymers (Table 2). The highest formaldehyde content is found in GUMEFA produced without a plasticizer. In contrast, for GUMEFA samples made with HEDP, the formaldehyde content remains almost unchanged and falls within the margin of error (0.02 wt.% with the SF method, Table 2).

## 4. Conclusions

In this study, a glycoluryl and melamine complex (GU-ME) at a 1:5 ratio was synthesized and characterized using IR and NMR spectroscopy. In the next phase, this complex was polycondensed into the polymer GUMEFA using HEDP as a “green” plasticizer, and its formation chemistry was proposed. The resulting GUMEFA was analyzed for its free formaldehyde and methylol group contents. It was found that GUMEFA produced with HEDP contained lower amounts of free formaldehyde (1.15–1.34 wt.%) and methylol groups (1.56–0.54 wt.%) compared with the resin plasticized with HCl. These results indicate that using HEDP as a plasticizer reduces the free formaldehyde levels and broadens the potential applications of the resulting GUMEFA. The study enhances the understanding of the plasticization process for glycoluril–melamine resins and allows for the optimization of the production process to minimize free formaldehyde, thereby increasing the range of possible applications. Additionally, the findings will assist in developing resins with various compositions and purposes, paving the way for creating composite materials with desired properties.

## Figures and Tables

**Table 1:** Formaldehyde content in samples obtained using spectrophotometric (SP) and fluorimetric methods (FL).

Sample	Plasticizer	Method	X_avg_, %
Sample 1	HCl conc.	SP	1.29
Sample 1	HCl conc.	FL	1.40
Sample 2	HEDP cr.	SP	1.26
Sample 2	HEDP cr.	FL	1.34
Sample 3	HCl dil.	SP	1.15
Sample 3	HCl dil.	FL	1.28
Sample 4	HEDP sol.	SP	1.24
Sample 4	HEDP sol.	FL	1.22
Sample 5	Without plasticizer	SP	1.53
Sample 5	Without plasticizer	FL	1.62

**Table 2:** Results of determination of methylol groups and formaldehyde using reverse iodometric titration.

Plasticizer	Content of Methylol Groups and Formaldehyde, %
HCl conc.	24.7
HEDP cr.	2.9
HCl dil.	15.5
HEDP sol.	1.7
Without plasticizer	2.7

## Data Availability

The original contributions presented in this study are included in this article. Further inquiries can be directed to the corresponding authors.
